# Patient Satisfaction and Their Willingness to Pay for a Pharmacist Counseling Session in Hospital and Community Pharmacies in Saudi Healthcare Settings

**DOI:** 10.3389/fphar.2020.00138

**Published:** 2020-03-02

**Authors:** Dhfer Mahdi AlShayban, Atta Abbas Naqvi, Md. Ashraful Islam, Mohammed Almaskeen, Ali Almulla, Muhab Alali, Abdullah AlQaroos, Mohamed Raafat, Muhammad Shahid Iqbal, Abdul Haseeb

**Affiliations:** ^1^ Department of Pharmacy Practice, College of Clinical Pharmacy, Imam Abdulrahman Bin Faisal University, Dammam, Saudi Arabia; ^2^ College of Clinical Pharmacy, Imam Abdulrahman Bin Faisal University, Dammam, Saudi Arabia; ^3^ Department of Pharmacology & Toxicology, College of Pharmacy, Umm Al Qura University, Makkah, Saudi Arabia; ^4^ Department of Clinical Pharmacy, College of Pharmacy, Prince Sattam Bin Abdul Aziz University, Alkharj, Saudi Arabia; ^5^ Department of Clinical Pharmacy, College of Pharmacy, Umm Al Qura University, Makkah, Saudi Arabia

**Keywords:** patient satisfaction, counseling, willingness to pay, health services, community pharmacy service, hospital pharmacy service, pharmacoeconomics, cost benefit analyses

## Abstract

**Objective:**

Patient satisfaction is an indicator for quality of healthcare service and is sometimes linked to patients’ willingness to pay. Willingness to pay is an economic method for estimating patient’s inclination for a service in monetary terms. This study assessed satisfaction of patients from pharmacist counseling service and estimated their willing to pay for the same.

**Methods:**

A month-long survey was conducted in community and hospital pharmacies located in Khobar, Dammam, and Qatif cities of Saudi Arabia, using Arabic version of Patient Satisfaction Feedback (PSF) questionnaire that measured satisfaction with counseling as well as willingness-to-pay. Convenient sampling method was used, and sample size was calculated based on power analysis. Data was analyzed through SPSS version 23. Chi-square (χ^2^) test and logistic regression analyses were conducted to report associations between variables and, determinants of satisfaction as well as willingness to pay respectively. The study was approved by concerned ethical committee (IRB-2019-05-020).

**Results:**

Patients (n = 531) with previous counseling experience were more likely to be satisfied [adjusted odds ratio (AOR) 5.2, p < 0.05]. Patients were more willing to pay if, they had an income above SAR 10,000 i.e., USD 2666.5 (AOR 1.78, p < 0.05), were satisfied with counseling time duration (AOR 4.5) and, were able to get counseling without difficulty (AOR 2.1, p < 0.05). Patients were more likely to be satisfied and were willing to pay if, they received required knowledge/information completely (AOR 2.5, 3.7, and p < 0.05) and found pharmacist helpful (AOR 1, 4.5, and p < 0.05). Most patients (43.9%) were satisfied with pharmacist counseling and average satisfaction rating was 7.87 ± 1.99/10.

**Conclusion:**

Patients considered counseling as an important service and were satisfied from it. Less than a third of patients were willing to pay for the service. Knowledge and helpfulness of pharmacist were identified as two major determinants that could not only satisfy and but also promote willingness to pay for the service. A pharmacist with skills in pharmaceutical care and counseling could be useful in promoting the service and making it profitable for pharmacy business.

## Background

Patient satisfaction is one of the most common and indirect indicators for evaluating the quality of health service (Prakash, 2010). Available evidence indicates that patients who are satisfied from a healthcare service are more likely to achieve their target goals of therapy (Tabbish, 2001). From a patient’s perspective satisfaction could translate into empowerment in clinical decision making, promote a positive attitude toward health and medicines taking behavior, as well as better disease state management (Wendy and Scott, 1994; Foot, 2004). Besides, satisfied patients are more likely to have better recovery that motivates healthcare provider to deliver better care (Wendy and Scott, 1994; Naqvi et al., 2019). From an organization’s perspective, it is an important for sustaining business as economic analyses related to patient satisfaction estimate a figure of USD 0.2 million as a lifetime income loss to the healthcare facility due to patient discontent (Voluntary Hospitals of America, 1988; Naqvi et al., 2019). In healthcare system of France and Germany, measuring satisfaction of patients from healthcare delivery is required for quality assurance (Boyer et al., 2006; Schoenfelder et al., 2011). On a positive side, satisfaction fosters patient loyalty that results in better patient retention (Pavel et al., 2015). The healthcare facility may receive more recommendations and subsequently increase its profits. Moreover, studies mention that satisfied patients were willing to pay for the service (Poulas et al., 2008; Pavel et al., 2015).

Willingness to pay (WTP) is a pharmacoeconomic evaluation technique to understand how important patients consider the service and prefer it. It is a type of cost-benefit analysis (CBA) that measures the benefit of a health service and cost of service in monetary terms (Johannesson and Jonsson, 1991). In health utility and welfare economics, the WTP is the amount in monetary value that a patient would be ready to spend to utilize a healthcare service (Mitchell & Carson, 1989; Donaldson, 1990). The WTP method is by far the most suitable method to measure the economic value of un-marketed services such as counseling in this case (Smith, 1983; Johannesson and Jonsson, 1991; O’Brien and Viramontes, 1994; O’Brien et al., 1998; Suh, 2000). This could be examined by utilizing several methods namely conjoint analysis, choice modeling, discrete choice experiment (DSE), or the most commonly used contingent valuation (CV) technique, with either revealed or stated preference approach in which patients are asked to quote an amount that they are willing to pay for a particular service (Suh, 2000; Taylor and Armour, 2002; Drummond et al., 2005; Aizuddin et al., 2014; Gupta and Çakanyıldırım, 2016; Lakić et al., 2017). Few studies have endeavored to measure the economic impact of pharmacy services through WTP as a proxy (Smith, 1983; Johannesson et al., 1993; Gafni, 1997; Suh, 2000).

Studies have reported a range of 13–57% of patients who indicated their willingness to pay for various pharmacy services (Smith, 1983; Larson, 2000; Suh, 2000). It was reported in a study that more than half of patients were willing to pay an average USD 23 for one-time pharmaceutical care consultation by pharmacist. A range of USD 4–40 was reported in literature as the amount patients were willing to pay for pharmacist consultations (Shafie and Hassali, 2010). Available evidence suggests that WTP is affected by patients’ demographic factors such as age, health and well-being, monthly family income, provision of medical insurance, as well as previous experience with the service (Einarson et al., 1988; Reutzel and Furmaga, 1993; Gore and Madhavan, 1994; Suh, 2000). Studies that evaluate WTP for pharmacy counseling service in developing countries are scant. A study by Naqvi and colleagues reported that 56% of Pakistani patients appeared willing to pay an average USD 3 as fee for a one-time counseling session with pharmacist (Naqvi et al., 2019). Another study by Shafie and Hassali reported that 67% of Malaysian patients indicated their willingness to pay an average USD 2.86 for a dispensing service by pharmacist (Shafie and Hassali, 2010). Moreover, a study by Alhaddad in Western region of Saudi Arabia revealed that 70% of patients were willing to pay an average USD 16 for pharmacist led medication therapy management service (Alhaddad, 2019).

Apart from the work of Pavel and colleagues, there is no study that has measured satisfaction of patients together with their willingness to pay for the service (Pavel et al., 2015). Moreover, data from the developing countries are lacking as none of the studies had measured patients WTP for a counseling service. The Malaysian and Saudi studies did not measure the WTP for counseling service (Shafie and Hassali, 2010; Alhaddad, 2019). Though, Naqvi and colleagues measured WTP for pharmacist counseling however, the demographics of Pakistani and Saudi patients were quite different. Hence, they could not be compared since demographic characteristics may act as determinants of WTP. Therefore, there was a need to evaluate patients’ satisfaction and WTP for pharmacist counseling in Saudi healthcare settings.

## Methods

### Study Design and Settings

This cross-sectional study was carried out during March 2019 to April 2019. It was conducted in hospital and community pharmacies located in three cities namely Khobar, Dammam, and Qatif, that are situated in Eastern Province of Saudi Arabia. The study targeted patients who were counseled by pharmacists.

### Objective

The aim was to assess patient satisfaction from counseling provided by pharmacists and their willingness to pay (WTP) for the same.

### Study Venues

**Figure 1:**
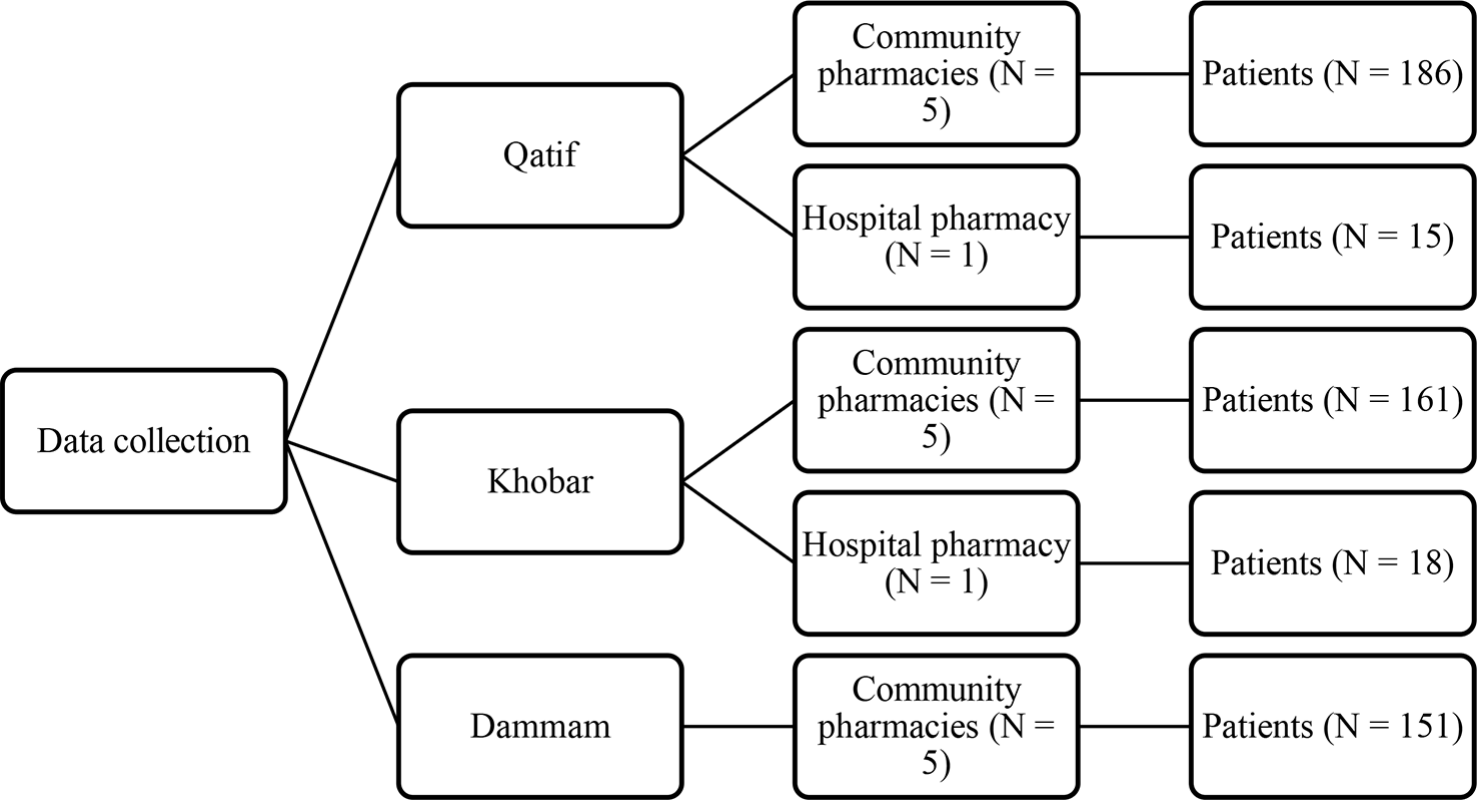
Study venues and patient enrollment.

The venues of the study were hospital and community pharmacies. Hospital pharmacies of King Fahd Hospital of the University located in city of Khobar and, Al Zahra Hospital in Qatif city, as well as community pharmacies located in cities of Khobar, Dammam, and Qatif served as venues. All the three cities are located in Dammam Region that forms the third most populated metropolitan region of Saudi Arabia. The major city of this region is Dammam which is also the Governorate of the province. The city of Khobar is the second most populated city in the province. Selection of hospital pharmacies was made to sample a representative population of Saudi patients. Community pharmacies were located in every suburb of the cities in this region. Hence, each city was hypothetically divided into five zones namely north, south east, west, and central. One community pharmacy was selected as a venue from each hypothetical zone of the cities (**Figure 1**).

### Eligibility Criteria

The study included adult male and female patients who spoke either Arabic or English. Patients aged 18 years or older were included. Patients with an acute or chronic illness, who had a counseling session with pharmacist were invited to participate. Patients who did not fill the above-mentioned criteria were not included. Patients who did not consent to participate and those who returned incomplete questionnaires were left out. Pharmacists with a bachelor’s degree, registered with the country’s regulatory body and, with at least 1-year work experience were included in the study.

### Data Collection and Counseling Session

The data collection in hospital pharmacies was carried out in evening hours on weekdays. Additionally, data collection in community pharmacies was carried out on weekends (Friday and Saturday) in evening. The selection of these timings was based on peak patient visiting hours. The pharmacist counseling session was a single face-to-face oral communication session with patient. The counseling was based on patient’s prescription related queries, disease and therapy education, or any other query the patient had. The average duration of the session was around 8 min.

### Research Tool and Translation

We used the patient satisfaction feedback (PSF) questionnaire to collect data. It was developed by Naqvi and colleagues in Urdu and English languages. The PSF is a 10-item questionnaire that contains questions related to patients’ satisfaction and WTP for counseling session with pharmacist. All items of the scale except item 8 and 10, are multiple choice. Item 8 is related to WTP and is open-ended. A respondent is free to mention any amount he/she is willing to pay considering if the counseling is a charged service. Item 10 is in the form of a rating scale (Naqvi et al., 2019).

The tool was translated in Arabic language before use. The translation process was conducted based on standard guidelines for translating and cross-culture adaptation for questionnaires (Gjersing et al., 2010; Epstein et al., 2015). We used the forward and backward translation by bilinguals (Degroot et al., 1994). Two academicians whose first language was Arabic and English as second language, translated and prepared the first draft in Arabic language. Both academicians were blinded to each other. Subsequently, the two drafts were then compared, and any disagreements were sorted out at this point. An Arabic version of PSF questionnaire (PSF-AR) was finalized. This PSF-AR was then back-translated into English by a team comprising of a pharmacist, a linguist, and a professor. The pharmacist, linguist, and professor were unaware of the purpose of the tool. The Arabic version of PSF was a deemed suitable to use at this point.

### Piloting of the Translated Version

The Arabic version of the tool was piloted in a total of 58 patients and this process was conducted in two phases. The methodology of piloting was adopted from Converse and Presser (Converse and Presser, 1986). The first phase was participatory in which the questionnaire was given to 23 patients who were informed that the survey was a pre-test for the newly formulated Arabic version of the PSF. The patients were asked to fill the survey and were invited to give feedback. All patients found the instructions clear and no changes or modifications were proposed. The second phase was an undeclared survey and the questionnaire was handed to another 35 patients in a real-time scenario as a quality control feedback on their experience with pharmacist counseling. Participants filled the questionnaire after attending a counseling session with pharmacists. Attention was paid to identify any query raised by patients in filling their response. No difficulty in reading and understanding of PSF-AR was observed during this process. This two-tiered piloting process ensured robust pre-testing of questionnaire. The results of pilot study were not included in main findings.

### Sample Size and Sampling

Due to generalization of results over the population, sample size adequacy demand major concern. To determine sample size from unknown size of population, “power study” is a useful and frequently used tool in medical research. A total of 510 sample can achieve more than 90% power when 3% margin of error is considered. The size of sample was calculated using the following formula after collecting required information from the available literature (Naing et al., 2006).

$$n=\frac{{\left(Z_{1-\beta }\right)}^{2}\left[p(1-p)\right]}{d^{2}}$$

where, n is required sample size, Z_1-β_ is Z value at power 1-β (considered 95% power and the value is 1.64), p is the value for referred prevalence as retrieved from published article i.e., 44.4% (value is 0.444), d is the margin of error (at 3% the value is 0.03). Convenient sampling method was used to collect the samples and the collection procedure stopped after completing the required number of patients.

### Variable Management and Data Cleaning

Counseling satisfaction and willingness to pay were the two outcome variables for the current study. At the initial level, data of counseling satisfaction were collected using response of four categories (very satisfied, satisfied, uncertain, and not satisfied). For multivariate analysis satisfaction level was re-categorized into two groups, i.e., very satisfied and satisfied into, “satisfied” (coded, 1) and, uncertain and not satisfied into, unsatisfied (coded 0). To find the predictors of “willingness to pay” the dependent variable, “yes” was coded as 1 and, “no” as 0. Independent variables including patients’ demographics were coded and recoded as required. Data were checked and cleaned for missing cases, incomplete information, and also for extreme value using informal technique (Dunn and Clark, 1975; Islam et al., 2016).

### Data Analyses

IBM SPSS version 23 (IBM Corp. Armonk, NY, USA) was used to analyze the data. Descriptive statistics was used to describe patient demographic and the results were mentioned as frequency and their percentage (%). Continuous data was reported in mean (X) with standard deviation (SD), and median (M) with inter quartile range (IQR). Chi-square (χ^2^) test was used to select significant independent variables to run a multiple model. Logistic regression was conducted to determine the predictors of counseling satisfaction and WTP. The model fitness was tested using Hosmer and Lemeshow test, classification table, and Negelkerker R^2^. Magnitude of standard error (SE) can be an indicator of testing multicollinearity among the predictors in multiple model. For current study we considered the value of SE less than 2.0 indicating no evidence of multicollinearity (Chan, 2004). A two-tailed p value of 0.05 was considered significant at the 95% CI (confidence interval) level.

### Ethics Approval and Consent

All patients who agreed to participate in the study were briefed about the study objectives and were required to provide their written consent. Data was collected from those patients who provided their consent. The study was approved from the Institutional Review Board of Imam Abdulrahman Bin Faisal University, Dammam, Saudi Arabia (IRB-2019-05-020).

## Results

A total of 531 patients responded to the survey. Most responses were from patients who visited community pharmacy (N = 498, 93.8%). The mean age of patients was 34.3 ± 12.5 years ranging from 18 to 80 years. Three quarters of patients were adults (N = 400, 75.3%) and more than half were males (N = 324, 61%). Most patients were married (N = 369, 69.5%) and had secondary education (N = 223, 44.3%). Slightly more than half of patients were employed (N = 291, 54.8%) and had acute illness (N = 293, 55.2%). Majority had a monthly family income between SAR 5,000 and 10,000 (N = 209, 39.4%) (**Table 1**).

**Table 1:** Background characteristics of the study patients (n=531).

Characteristics	Frequency (%)	Mean (SD)/median (IQR)
***Demographic variables***		
Age		34.34 (12.55)
Adult	443 (83.4)	
Older	88 (16.6)	
Gender		
Male	324 (61.0)	
Female	207 (39.0)	
Marital status		
Single	162 (30.5)	
Married	369 (69.5)	
Education level		
No education	30 (5.6)	
Primary	42 (7.9)	
Secondary	235 (44.3)	
Graduation	224 (42.2)	
Occupation		
Unemployed	61 (11.5)	
Student	97 (18.3)	
Employed	291 (54.8)	
Household work	82 (15.4)	
Monthly income (SAR)		
Less than 5,000 (< USD 1332.8)	204 (38.4)	
Between 5,000 and 10,000 (USD 1,332.8–2,665.6)	209 (39.4)	
More than 10,000 (> USD 2,665.6)	118 (22.2)	
***Patients characteristics***		
Type of pharmacy		
Community pharmacy	498 (93.8)	
Hospital pharmacy	33 (6.2)	
Type of illness		
Acute illness	293 (55.2)	
Chronic illness	138 (44.8)	
Experienced pharmacist counseling before		
Yes	457 (86.1)	
No	74 (13.9)	

USD 1 equals SAR 3.75.

Regarding patient counseling information, most of the patients (N = 468, 88%) received counseling without any difficulty while less than half (N = 252, 47.6%) indicated that they received required information/knowledge to some extent. Almost similar proportion of patients (N = 257, 48.4%) indicated that they found the pharmacist somewhat helpful in resolving their queries. More than half of patients (N = 307, 57.8%) opined that appropriate time was given in counseling. They further mentioned that counseling service should be offered by pharmacies in localities (N = 491, 92.5%), and indicated that they would recommend others to seek counseling from pharmacists (N = 476, 89.6%). However, less than a third of patients (N = 154, 29%) indicated their willingness to pay for counseling service should it be charged, and an average of SAR 25 (USD 6.67) was indicated as possible fee payable by patients for one-time counseling session. Most patients (N = 233, 43.9%) were satisfied with pharmacist counseling and average satisfaction rating was 7.87 ± 1.99 out of 10 (**Table 2**).

**Table 2:** Descriptive statistics about patients counseling factors.

Factors	Frequency (%)	Mean (SD)/median (IQR)
Getting counseled without any difficulty		
Yes	468 (88.1)	
No	63 (11.9)	
Received required knowledge/information		
Completely	248 (46.7)	
To some extent	253 (47.6)	
No	30 (5.6)	
Pharmacists helpfulness		
Very helpful	246 (46.3)	
Somewhat helpful	257 (48.4)	
Not helpful	28 (5.3)	
Opinion about time duration		
More time should be given	193 (36.3)	
Appropriate time was given	307 (57.8)	
My time was wasted	31 (5.8)	
Recommendation to others for counseling		
Yes	476 (89.6)	
No	55 (10.4)	
Opinion about counseling service being offered in your locality		
Yes	491 (92.5)	
No	40 (7.5)	
Willingness to pay for counseling service		
Yes	154 (29.0)	
No	377 (71.0)	
Amount willing to pay for one-time counseling session (in SAR) (n=154)		25 (35)*
Satisfaction level		7.87 (1.99)
Very satisfied	228 (42.9)	
Satisfied	233 (43.9)	
Uncertain	55 (10.4)	
Not satisfied	15 (2.8)	

*Median with IQR.

The patient demographics and counseling factors were cross tabulated with their satisfaction and willingness to pay for counseling service using chi-square test for association. The association of “gender” with “willingness to pay,” was significant (p < 0.05). Moreover, association of “monthly family income” was significantly associated with “willingness to pay” (p < 0.05). No other demographic variable was significantly associated with willingness to pay. Furthermore, no significant association was observed in cross tabulation between demographics and satisfaction (p > 0.05).

In addition, the counseling factors were cross tabulated with the same to observe any association. There was a significant association between “satisfaction” and the variable of “getting counseled without difficulty” (p < 0.001). The counseling variable of “received required information/knowledge” was significantly associated with “patient satisfaction” (p < 0.001), and “willingness to pay” (p < 0.01). Similarly, significant association was reported for the variable of, “found pharmacist helpful,” with, “patient satisfaction” (p < 0.001), as well as, “willingness to pay” (p < 0.05). The counseling variables of “patient opinion about time of counseling,” “recommending counseling to others,” and, “should this service be offered in locality,” were significantly associated with “patient satisfaction” and p-values obtained were less than 0.001 (**Table 3**).

**Table 3:** Association of various demographic and counseling factors with patients’ satisfaction and willingness to pay for counseling.

Factors	Satisfied		Willing to pay	
	Observed count (expected count)			
	No	Yes	No	Yes
***Demographic factors***				
Age	ns		ns	
Adult	58 (20.8)	385 (90.2)	318 (70.8)	125 (31.3)
Older	12 (13.6)	76 (86.4)	59 (67.0)	29 (33.0)
Gender	ns		*	
Male	45 (13.9)	279 (86.1)	219 (67.6)	105 (32.4)
Female	25 (12.1)	182 (87.9)	158 (76.3)	49 (23.7)
Marital status	ns		**	
Single	27 (16.7)	135 (83.3)	130 (80.2)	32 (19.8)
Married	43 (11.7)	326 (88.3)	247 (66.9)	122 (33.1)
Education level	ns		ns	
No education	6 (20.0)	24 (80.0)	24 (80.0)	6 (20.0)
Primary	6 (14.3)	36 (85.7)	29 (69.0)	13 (31.0)
Secondary	23 (9.8)	212 (90.2)	169 (71.9)	66 (28.1)
Graduation	35 (15.6)	189 (84.4)	155 (69.2)	69 (30.8)
Occupation	ns		ns	
Unemployed	12 (19.7)	49 (80.3)	47 (77.0)	14 (23.0)
Student	13 (13.4)	84 (86.6)	73 (75.3)	24 (24.7)
Employed	37 (12.7)	254 (87.3)	193 (66.3)	98 (33.7)
Household work	8 (9.8)	74 (90.2)	64 (78.0)	18 (22.0)
Monthly income (SAR)	ns		*	
Less than 5,000 (< USD 1332.8)	22 (10.8)	182 (89.2)	148 (72.5)	56 (27.5)
Between 5,000 and 10,000 (USD 1332.8–2,665.6)	26 (12.4)	183 (87.6)	157 (75.1)	52 (24.9)
More than 10,000 (> USD 2665.6)	22 (18.6)	96 (81.4)	72 (61.0)	46 (39.0)
***Patients characteristics***				
Type of pharmacy	ns		***	
Community pharmacy	68 (13.7)	430 (86.3)	364 (73.1)	134 (26.9)
Hospital pharmacy	2 (6.1)	31 (93.9)	13 (39.4)	20 (60.6)
Type of illness	ns		ns	
Acute illness	42 (14.3)	251 (85.7)	216 (73.7)	77 (26.3)
Chronic illness	28 (11.8)	210 (88.2)	161 (67.6)	77 (32.4)
***Counseling factors***				
Experienced pharmacist counseling before	*		ns	
Yes	54 (11.8)	403 (88.2)	326 (71.3)	131 (28.7)
No	16 (21.6)	58 (78.4)	51 (68.9)	23 (31.1)
Getting counseled without difficulty	***		ns	
Yes	43 (9.2)	425 (90.8)	337 (72.0)	131 (28.0)
No	27 (42.9)	36 (57.1)	40 (63.5)	23 (36.5)
Received required knowledge/information	***		**	
Completely	4 (1.6)	244 (98.4)	160 (64.5)	88 (35.5)
To some extent	38 (15.0)	215 (85.0)	192 (75.9)	61 (24.1)
No	28 (93.9)	2 (6.7)	25 (83.3)	5 (16.7)
Pharmacists helpfulness	***		*	
Very helpful	2 (0.8)	244 (99.2)	162 (65.9)	84 (34.1)
Somewhat helpful	44 (17.1)	213 (82.9)	191 (74.3)	66 (25.7)
Not helpful	24 (85.7)	4 (14.3)	24 (85.7)	4 (14.3)
Opinion about counseling time duration	***		ns	
More time should be given	19 (9.8)	174 (90.2)	137 (71.0)	56 (29.0)
Appropriate time was given	26 (8.5)	281 (91.5)	219 (71.3)	88 (28.7)
My time was wasted	25 (80.6)	6 (19.4)	21 (67.7)	10 (32.3)
Recommendation to others for counseling	***		ns	
Yes	35 (7.4)	441 (92.6)	334 (70.2)	142 (29.8)
No	35 (63.6)	20 (36.4)	43 (78.2)	12 (21.8)
Opinion about counseling service being offered in your locality	***		ns	
Yes	45 (9.2)	446 (90.8)	347 (70.7)	144 (29.3)
No	25 (62.5)	15 (37.5)	30 (75.0)	10 (25.0)
Willingness to pay for counseling service	ns		NA	
Yes	15 (9.7)	139 (90.3)		
No	55 (14.6)	322 (85.4)		
Satisfaction level	NA		ns	
Not satisfied			55 (78.6)	15 (21.4)
Satisfied			322 (69.8)	139 (30.2)

chi-square test * = p < 0.05; ** = p < 0.01; *** = p < 0.001, ns, not significant, NA, not applicable, USD 1 equals SAR 3.75.

**Figure 2:**
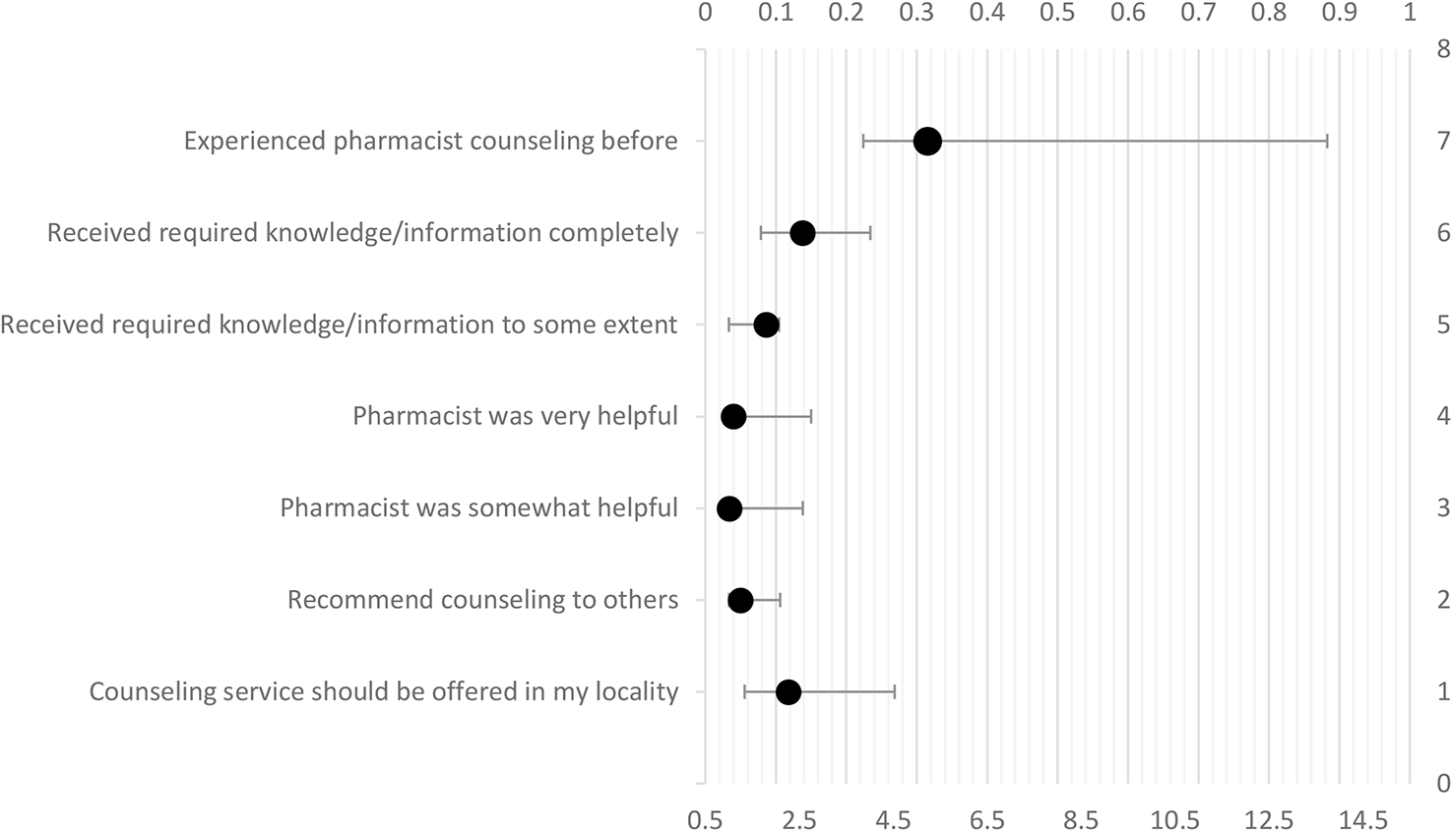
Model for satisfaction with pharmacist counseling.

Logistic regression revealed that patients who previously had counseling sessions with pharmacists were five times more likely to be satisfied [adjusted odds ratio (AOR) = 5.2, p < 0.05] compared to patients who never had counseling before. Patients who found pharmacist helpful were more likely to be satisfied (AOR = 1.09, p < 0.001) compared to patients who did not find them helpful, patients who completely received required information/knowledge from pharmacist were 2.5 times more likely to be satisfied (AOR = 2.563, p < 0.001). Besides, patients who indicated that they received required information/knowledge to some extent, were 1.8 times more likely to be satisfied (AOR = 1.789, p < 0.01). Patients who opined that counseling time was appropriate, were 4.5 times more likely to be satisfied (AOR = 4.521, p < 0.05) compared to those who considered counseling as a waste of time. Patient who recommended counseling to others and those who opined that the counseling service should be offered in pharmacies in their locality, were more likely to be satisfied (AOR = 1.245, p < 0.01) as compared to patients who did not. The model for patient satisfaction is tabulated in **Table 4** and represented graphically in **Figure 2**. The model is adjusted for demographic variables namely age, education, occupation, and income. In multiple logistic regression, “Enter” method was applied, multicollinearity was checked and was not found. Hosmer-Lemeshow test value was χ2 = 2.444, p = 0.964, Pearson chi-square, significance for model at p < 0.001, and classification table (overall correctly classified percentage = 86.8), were applied to check the model fitness. Cox & Snell R Square was 0.396 while Nagelkerke R Square was reported at 0.732 (**Table 4**; **Figure 2**).

**Table 4:** Model for satisfaction.

Variables	B	SE	p-value	AOR	95% CI for AOR	
					Lower	Upper
Experienced pharmacist counseling before
Yes	1.695	0.920	0.037	5.230	3.862	13.742
No ^®^						
Getting counseled without difficulty
Yes	0.869	0.590	0.141	2.237	0.683	7.330
No ^®^						
Received required knowledge/information
Completely	0.941	0.869	<0.001	2.563	1.683	4.014
To some extent	0.582	0.642	0.001	1.789	1.001	2.071
No ^®^						
Pharmacists helpfulness
Very helpful	0.091	0.036	<0.001	1.095	1.001	2.752
Somewhat helpful	0.005	0.001	0.016	1.005	1.004	2.575
Not helpful ^®^						
Opinion about time of counseling duration
More time should be given	1.939	0.976	0.136	6.954	0.621	47.915
Appropriate time was given	1.509	1.103	0.047	4.521	1.026	32.129
My time was wasted ^®^						
Recommendation to others for counseling
Yes	0.219	0.554	<0.001	1.245	1.004	2.093
No ^®^						
Opinion about counseling service being offered in your locality
Yes	0.817	0.705	0.004	2.263	1.336	4.531
No ^®^						
Willingness to pay for counseling service
Yes	0.282	0.521	0.588	1.325	0.272	2.093
No ^®^						

R, reference case, CI, confidence interval; AOR, adjusted odds ratio.

Similarly, multiple logistic regression for WTP adjusted for demographic variables age, gender, education, and occupation, was applied using “Enter” method. Multicollinearity was checked and was not present. The value for Hosmer-Lemeshow test was reported at χ2 = 7.452, p=0.489. Pearson chi-square, significance for model at p < 0.01, and classification table (overall correctly classified percentage = 72.5) were applied to check the model fitness. Cox & Snell R Square value was 0.172 while Nagelkerke R Square was reported at 0.209.

**Figure 3:**
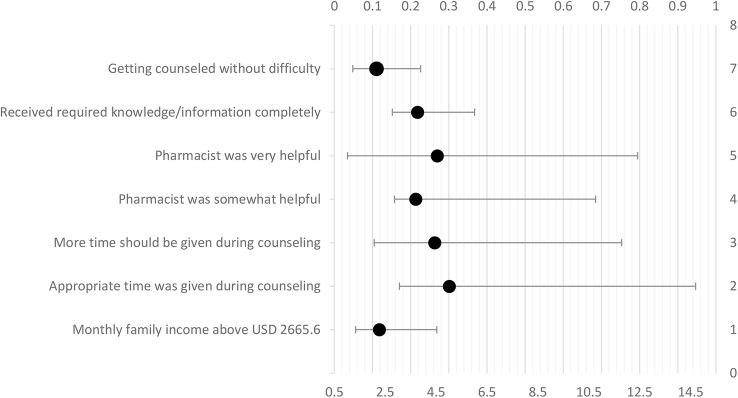
Model for willingness to pay.

Patients who had counseling without experiencing any difficulty were 2.16 times more likely to pay (AOR = 2.164, p < 0.05) as compared to patients who had difficulties in getting counseled. Patients who completely received required information/knowledge from pharmacist counseling appeared more likely to pay for the service (AOR = 3.76, p < 0.05) as compared to patients who did not. Patients who found pharmacist very helpful and somewhat helpful in resolving their queries appeared more likely to pay (AOR = 4.54, and 3.69 respectively; p < 0.05) as compared to patients who did not. Patients who opined that more time should be given for counseling appeared more likely to pay (AOR = 4.45, p < 0.05) as compared to patients who opined that their time was wasted in counseling. For patients who mentioned that appropriate time was given in counseling session, had higher odds (AOR = 5.017) of paying for counseling. Patients who had higher income were more likely to pay for counseling (AOR 1.78, p < 0.05). The model is presented in **Table 5** and **Figure 3**.

**Table 5:** Model for willingness.

Variables	B	SE	p-value	AOR	95% CI for AOR	
					Lower	Upper
Experienced pharmacist counseling before
Yes	0.052	0.308	0.865	1.053	0.504	1.675
No ^®^						
Getting counseled without difficulty
Yes	0.772	0.346	0.022	2.164	1.230	3.893
No ^®^						
Received required knowledge/information
Completely	1.318	0.718	0.031	3.763	2.782	6.014
To some extent	0.832	0.694	0.234	2.299	0.638	9.560
No ^®^						
Pharmacists helpfulness
Very helpful	1.513	0.762	0.047	4.542	1.020	12.423
Somewhat helpful	1.307	0.740	0.039	3.695	2.867	10.772
Not helpful ^®^						
Opinion about time of counseling duration
More time should be given	1.492	0.626	0.017	4.445	2.069	11.797
Appropriate time was given	1.613	0.618	0.009	5.017	3.062	14.704
My time was wasted ^®^						
Recommendation to others for counseling
Yes	0.318	0.434	0.465	1.374	0.568	3.221
No ^®^						
Opinion about counseling service being offered in your locality
Yes	0.189	0.475	0.690	1.208	0.327	2.094
No ^®^						
Satisfaction with counseling service
Yes	0.280	0.484	0.563	1.323	0.293	1.952
No ^®^						
Monthly family income (SAR)
Less than 5,000 ^®^						
Between 5,000 and 10,000	-0.125	0.238	0.598	0.882	0.553	1.406
More than 10,000	0.579	0.289	0.045	1.784	1.012	3.144

R, reference case, AOR, adjusted odds ratio, CI, confidence interval, SAR, Saudi Arabian riyal.

## Discussion

Patient satisfaction is regarded as an indirect indicator of quality of healthcare service. It has the potential to translate into better patient care and as well as increase profitability for the service provider. Studies mention that satisfaction increases patients’ willingness to pay for service. This is important considering marketable potential of service. Our study documented patient satisfaction and their willingness to pay for counseling, as a pharmaceutical service provided at hospital and community pharmacies. With the exception of the work of Alhaddad, no study has been conducted in Saudi Arabia that reports this phenomenon (Alhaddad, 2019). This study was conducted in the third largest metropolitan region of Saudi Arabia comprising of three major cities with a population of over 0.3 million (General Authority for Statistics, 2016; AlQarni et al., 2019). The study utilized a previously validated instrument known as patient satisfaction feedback (PSF) that was translated in Arabic language, and gathered responses from a large patient sample in both hospital and community pharmacy settings (Naqvi et al., 2019). These are some notable strengths of this work.

The study reported that slightly less than half of the patients (43.9%) were satisfied while 42.9% of patients indicated that they were “very” satisfied with counseling. This indicated that about 87% of patients were content with the service. This figure for satisfaction was quite high compared to previous studies reported from other parts of the world. Yang and colleagues conducted a study in South Korean patients to report satisfaction with medication counseling provided in community pharmacies. They reported that a third of patients (34%) were satisfied (Yang et al., 2016). In a study in the Netherlands, Geffen and colleagues reported that less than half of patients (42%) were satisfied with medication counseling. The authors of that study concluded that patients’ expectations may not have been met (van Geffen et al., 2011). Another study in Pakistan reported that 60% of patients appeared satisfied with counseling (Naqvi et al., 2019). All studies except the one in Pakistan, were conducted in community pharmacies where the majority of patients appeared unsatisfied. The Pakistani study was based in hospital pharmacy settings where most patients appeared satisfied.

The present study included patients from both hospital and community pharmacy settings and based on the cross-tabulation results, there was no significant (p > 0.05) association between healthcare settings and satisfaction. A possible explanation to this occurrence in our study could be based on patient population. Patients who have had an experience with counseling before would be more acquainted with the service compared to those who have not had counseling before. In this study, most patients (86%) had previously attended some sort of counseling by pharmacists. Logistic regression analysis highlighted that such patients were more likely to be satisfied as compared to those who had no previous exposure. This finding is in line with results of Naqvi and colleagues (Naqvi et al., 2019). The hypothesis is further strengthened by findings of the study in Netherlands where most patients who were dissatisfied had never attended counseling sessions before (van Geffen et al., 2011). In addition, there was no significant (p > 0.05) association of demographic characteristics such as education, type of illness, and income, with satisfaction. This meant that educated and uneducated patients have an equal chance of being satisfied and *vice versa*. This highlights that patients may have expectations regarding counseling that are not dependent on patients’ level of education. Such expectations could be related to knowledge of disease and treatment.

A benchmark could be the work of Naqvi and colleagues where significant (p < 0.05) association of satisfaction was reported with type of illness and health insurance. This may be due to expectation of patients with chronic and acute illnesses. Pakistani patients with chronic illnesses seemed more likely to be satisfied with counseling compared to those with acute illnesses. Whereas satisfaction of Saudi patients was not associated with type of illness. This could be due to number of patients and time duration of counseling. Pakistan has a population of over 200 million while Saudi population is just over 30 million (General Authority for Statistics, 2016). Secondly, there are about 32,500 registered pharmacists in Pakistan while registered pharmacists in Saudi Arabia are about 24,395 (Naqvi et al., 2017; AlRuthia et al., 2018). Therefore, pharmacy workforce density in Saudi healthcare system is higher as compared to Pakistan. Hence, pharmacists in Saudi healthcare system can afford to give more time to patients during counseling (Alfadl et al., 2018). This hypothesis is further strengthened by our study findings regarding time duration of counseling, in which more than half of patients (58%) indicated that counseling time duration was “appropriate” whereas less than half of patients (42%) mentioned the same in Pakistani study (Naqvi et al., 2019). Therefore, patients with severe illnesses may have been prioritized for counseling in Pakistan.

Further, there was no significant association of satisfaction with income in Saudi patients. This occurrence could be explained based on out-of-pocket expenditure. Unlike patients in Pakistan, Saudi patients do not have to pay direct costs for treatment. Therefore, income is not a determinant of satisfaction. Patients of developing countries may have to bear out-of-pocket cost to purchase medicines. Though, Pakistani study reported no significant association of income with satisfaction, there was a significant association for availability of “health insurance” (Naqvi et al., 2019). This meant that Pakistani patients who had health insurance were more likely to be satisfied with counseling as they did not have to pay out-of-pocket costs.

The second parameter to observe while documenting the satisfaction was the quality of service. Patients satisfaction was significantly (p < 0.05) associated with, having counseling without facing difficulties, received required information and finding the pharmacist helpful. In our study patients who indicated that they found the pharmacist helpful in resolving their queries and had received required information/knowledge completely, were more likely to be satisfied. Yang and colleagues mentioned the attitude of pharmacist, use of patient-intelligible language and simpler information contents, as determinants of patient satisfaction (Yang et al., 2016). Considering the advancements in pharmacy profession, pharmacists have to assume the role of patient care provider and a member of allied healthcare team alongside traditional job responsibilities of medication dispensing and drug information services (Alamri et al., 2017). This could increase workload and subsequently add to the job stress that has the potential to decrease work efficiency of pharmacist. As a result, patients may be dissatisfied (Lea et al., 2012; Yang et al., 2016). Higher job stress levels related to patient care have been reported from pharmacists working in Saudi healthcare settings (Suleiman, 2015). Appropriate number of staff and effective time management could help pharmacists manage their traditional and clinical roles. This aspect is of paramount importance to pharmacy human resource as proper staffing would ensure better performance that could result in increased satisfaction from service and translate into business profitability (León et al., 2001; McIntosh et al., 2011).

There was a significant association between counseling time and satisfaction. Patients who mentioned that counseling time was appropriate were four times more likely to be satisfied and five times more likely to pay. Insufficient counseling time has been observed to be a determinant of patient dissatisfaction as studies indicate that counseling time >1 min has more potential to satisfy patients as compared to counseling session lasting less than a minute. Additionally, there may be a direct relationship between counseling duration and patient satisfaction (Yang et al., 2016; Naqvi et al., 2019). Moreover, less time duration of counseling session was observed to be the single most common predictor of dissatisfaction among South Korean patients (León et al., 2001; Lee et al., 2009; Yang et al., 2016).

The patients were asked about their willingness to pay for the counseling service should it be charged. Regression analysis indicated that satisfied patients were 1.3 times more likely to pay for the service. However, less than a third of patients (29%) indicated that they were willing to pay and declared an average SAR 25, i.e., USD 6.67, as fee for a one-time pharmacist counseling session. This amount was higher than that reported from Pakistani patients, i.e., USD 3, and, an American study in which patients were willing to pay USD 5.6 for pharmacist’s consultation (Suh, 2000; Naqvi et al., 2019). This is an indication of patients’ satisfaction and their inclination toward pharmacist counseling service. Evidence indicates that despite being satisfied, most patients are not willing to pay for pharmacy services and are likely to avail such extended pharmacy services only if it is available free of cost (Lee et al., 2009). The proportion of patients willing to pay for pharmacist counseling service in this study were lower than the figures reported in the literature, i.e., 31–45% (Smith, 1983; Laverty, 1984; Metge et al., 1998). WTP was significantly associated (p < 0.05) with patients’ monthly family income that implied that most patients with an income greater than SAR 10,000, i.e., USD 2,666.5, were 1.7 times more likely to pay for the service. This is in line with previous studies as WTP may depend on consumer’s purchasing power (Donaldson, 1999; Suh, 2000).

Consumer buying power is the capacity of the individual to buy a certain quantity of goods/services. A high value would indicate that the consumer has a higher income and power to purchase goods/service relative to the supply and economic value of goods/service available (Offodile and Ho, 2018). Therefore, patients with a high purchasing power, i.e., higher income relative to economic value of pharmacist counseling service, would be in a better position to utilize and pay for the service. Furthermore, willingness to pay was significantly associated (p < 0.05) with pharmacy settings. Patients who attended counseling session in hospital settings were more likely to pay as compared to those who attended the same in community pharmacy settings. Studies have mentioned that achievement of positive health outcomes are linked to WTP. Evidence indicates that patients would be willing to pay if their satisfaction with the following three healthcare features is increased. These are namely, relationship with healthcare professionals, fulfillment of patient’s medicines needs and, increased likelihood of recovery (Pavel et al., 2015). Since these three attributes are more likely to be achieved in a hospital setting, the patients are more likely to be willing to pay in those settings.

The variable of previous counseling experience was not significantly associated (p > 0.05) with WTP. Previous counseling experience may have increased their knowledge and have empowered them in managing their conditions. Hence, they are satisfied and may not require a paid session with pharmacist anymore. The association of satisfaction with previous counseling experience can also be explained by this occurrence. The patients who received counseling without facing any difficulty such as understanding of language, voice and speech, were 2.16 times more likely to pay for the session. Besides, patients who received required knowledge/information were 2.29–3.76 times more likely to pay for the service. Moreover, patients who indicated that they found the pharmacist useful were 3.69–4.54 times more willing to pay. These factors highlight the importance of acquiring skills for pharmaceutical care and patient counseling. A skilled pharmacist has more potential to not only promote patient satisfaction and WTP for the service but also to increase the likelihood of achieving positive health outcomes. Based on the Common-Sense Model of Illness Representation (CSM), a patient’s perception of health risk and the emotional response associated with the risk would define patient’s overall response to illness (French and Weinman, 2008). The response would affect the outcome of illness. The perception of illness is based on disease identity, timeline, consequences, cause, controllability, and emotionality (McAndrew et al., 2008; Huston and Houk, 2011; Cameron and Leventhal, 2012). A pharmacist with pharmaceutical care and counseling skills could provide disease education and conditioning that may empower patients in all six domains. As a result, patients with a positive perception of disease and controlled emotional response to the risks associated with negative outcomes of the disease, would be in a better position to achieve positive health outcomes.

The study had a limitation of strategic bias. Some patients may not have liked the questions related to WTP and subsequently chose to not answer them. This was not because they did not perceive the value of the service but felt that counseling service should be free of charge. Besides, there was no question that could identify the reasons behind their answer regarding WTP and their declared amount. Moreover, the selected sampling technique was convenient sampling. Therefore, readers are instructed to generalize the results with caution.

## Conclusion

This study highlighted that patients considered pharmacist counseling as an important service that could help in effective disease state management. The quality of counseling offered to patients in hospital and community pharmacies of this region was according to the expectations as most patients were satisfied from it. Contingent valuation method was useful to measure the value of pharmacist counseling service in monetary terms. Though, less than a third of patients were willing to pay for the service, we were able to identify the determinants that prompted patients to pay. The results of the study are useful in practical context if the determinants of WTP are considered while counseling patients. Knowledge and helpfulness of pharmacist were identified as two major determinants that could not only satisfy but make patients willing to pay for the service. A pharmacist with skills in pharmaceutical care and counseling could be useful in promoting the service thereby making it profitable for pharmacy business.

## Author’s Note

This research paper is based on student research project undertaken as thesis for partial fulfillment of Doctor of Pharmacy (Pharm.D) degree by MoA (#2150008886), AlA (#2150000700) and MuA (#2150003205), at College of Clinical Pharmacy, Imam Abdulrahman Bin Faisal University, Dammam 31441, Saudi Arabia. A research abstract based on initial preliminary findings related to patient satisfaction was presented as a poster in 1^st^ Knowledge, Skills, Ability in Pharmacy & Toxicology (KSAPT) Conference held on 4^th^ April 2019 in Al Khobar. Another research abstract detailing complete analysis related to satisfaction and WTP was presented as a poster in SIPHA 2020 that took place from January 21 to 23, 2020 at Riyadh, Saudi Arabia.

## Data Availability Statement

The raw data supporting the conclusions of this manuscript will be made available by the authors, without undue reservation, to any qualified researcher.

## Ethics Statement

The studies involving human participants were reviewed and approved by the Institutional Review Board of Imam Abdulrahman Bin Faisal University (IRB-2019-05-020). The patients/participants provided their written informed consent to participate in this study.

## Author Contributions

DA, AN, MAI, MoA, AlA and MuA conceived the idea and designed the study. AN wrote the proposal with DA, AH, MI, MR, MAI, MoA, AlA, AbA and MuA collected the data. DA, AbA, MR, MI and AH helped in translating the questionnaire and entered data in SPSS. DA, AN, and MoA wrote the abstract and introduction section. DA, AN, AlA, MuA and AbA wrote the methodology. MR, MAI, and AH wrote the discussion section. All authors provided their input in revision of the manuscript. All authors read and approved the final manuscript.

## Conflict of Interest

The authors declare that the research was conducted in the absence of any commercial or financial relationships that could be construed as a potential conflict of interest.

## Abbreviations

USD, United States dollar; WTP, willingness to pay; CBA, cost benefit analysis; PSF, patient satisfaction feedback; Pharm.D, doctor of pharmacy; PSF-AR, patient satisfaction feedback Arabic version; SPSS, Statistical Package for Social Sciences; SD, standard deviation; USA, United States of America; IQR, interquartile range; SE, standard error; CI, confidence interval; IRB, Institutional Review Board; SAR, Saudi Arabian riyal; AOR, adjusted odds ratio; CSM, common-sense model of illness representation.
